# Anticipating infectious disease re-emergence and elimination: a test of early warning signals using empirically based models

**DOI:** 10.1098/rsif.2022.0123

**Published:** 2022-08-03

**Authors:** Andrew T. Tredennick, Eamon B. O’Dea, Matthew J. Ferrari, Andrew W. Park, Pejman Rohani, John M. Drake

**Affiliations:** ^1^ Odum School of Ecology, University of Georgia, Athens, GA 30602, USA; ^2^ Center for the Ecology of Infectious Diseases, University of Georgia, Athens, GA 30602, USA; ^3^ Department of Infectious Diseases, University of Georgia, Athens, GA 30602, USA; ^4^ Western EcoSystems Technology, Inc., 1610 East Reynolds Street, Laramie, WY 82070, USA; ^5^ The Center for Infectious Disease Dynamics and Department of Biology, The Pennsylvania State University, University Park, PA 16802, USA

**Keywords:** critical slowing down, early warning signals, epidemiology, measles, infectious disease

## Abstract

Timely forecasts of the emergence, re-emergence and elimination of human infectious diseases allow for proactive, rather than reactive, decisions that save lives. Recent theory suggests that a generic feature of dynamical systems approaching a tipping point—early warning signals (EWS) due to critical slowing down (CSD)—can anticipate disease emergence and elimination. Empirical studies documenting CSD in observed disease dynamics are scarce, but such demonstration of concept is essential to the further development of model-independent outbreak detection systems. Here, we use fitted, mechanistic models of measles transmission in four cities in Niger to detect CSD through statistical EWS. We find that several EWS accurately anticipate measles re-emergence and elimination, suggesting that CSD should be detectable before disease transmission systems cross key tipping points. These findings support the idea that statistical signals based on CSD, coupled with decision-support algorithms and expert judgement, could provide the basis for early warning systems of disease outbreaks.

## 1. Introduction

Forecasts of the emergence and re-emergence of infectious diseases have the potential to save lives, money and human productivity by allowing for proactive, rather than reactive, preparedness measures [1]. Similarly, indicators of the elimination of infectious diseases can measure the effectiveness of ‘end game’ strategies aimed at disease eradication [2]. Predicting (re-)emergence and elimination is possible with parametric mathematical models of disease transmission, but their success relies on detailed understanding of the underlying transmission dynamics and adequate data [3]. We often do not have enough information (or time) to parametrize such models. An alternative approach is to use model-independent statistical signals that portend infectious disease (re-)emergence and elimination by detecting critical slowing down (CSD) as the system approaches a critical transition [4,5].

Emergence and elimination of an infectious disease both involve a critical transition, often reflected in deterministic models by a *transcritical bifurcation*, that occurs at the critical point where the effective reproduction number ($R_{e}$, corresponding to the number of secondary cases that arise from a single infected case in a population) is equal to one [6]. Thus, subcritical ($R_{e}<1$) and supercritical ($R_{e}>1$) systems represent alternative dynamical regimes [4,7,8].

Critical transitions in stochastic systems, such as systems of disease transmission, are often accompanied by CSD, a reduction in the resilience of a system to perturbations [9,10]. CSD can be measured by changes in the statistical properties of the system, often referred to as early warning signals (EWS), such as an increase in the variance and autocorrelation [7,11]. Recent theoretical work suggests that CSD occurs as disease dynamics approach $R_{e}=1$ from below (emergence) [4,12] and from above (elimination) [2,4,13], and that several EWS can anticipate the critical transition [14–16]. These findings suggest that such model-independent statistical signals could be operationalized as part of early warning systems for disease emergence and elimination, or re-emergence and outbreaks of endemic diseases.

Operationalizing such EWS, however, and deploying early warning systems based on them, face many challenges [1,17]. For example, using EWS in an ‘online mode’ requires choosing temporal windows over which EWS are calculated. These moving windows should be long enough to provide reliable statistics, but short enough to forget the past so as to not overwhelm information contained in new observations. Such fine-tuning is especially important for diseases that fluctuate seasonally, where EWS might always increase and then decrease over the course of the year, requiring computations to be reset each season [16]. Another challenge is defining thresholds for detection of an upcoming tipping point. Detection thresholds can be based on the absolute value of an EWS (e.g. warning if variance exceeds some value), the trend in an EWS over time (e.g. warning if the correlation of variance with time exceeds some value), or an algorithmic combination of many factors (e.g. variance and autocorrelation increases above some value several observation periods in a row). Brett & Rohani [18] pioneered the approach of developing algorithms for combining EWS, their values and their trends to best detect disease emergence and elimination, but much work remains to be done.

An important step in operationalizing EWS based on CSD is to stress test the performance of EWS in anticipating critical transitions. One way to stress test EWS is through empirical case studies: do EWS anticipate re-emergence and elimination in observed time series of disease incidence [19]? However, uncritical application of EWS to observed data could lead to researchers getting the right answer for the wrong reasons. EWS might perform well for a given time series, but for reasons having nothing to do with CSD [20–22]. Critical transitions may also occur in the absence of EWS [23]. Without knowing the critical point (i.e. when $R_{e}(t)=1$), it is impossible to know if EWS are in fact sending us the signal we think they are.

Another option is to use fitted models of disease transmission to test EWS. This offers several advantages. First, using a model to simulate time series of cases means one also has access to a time series of $R_{e}(t)$, essential for knowing the time at which the critical transition occurs. This means we know whether we are getting the right answer for the right reason. Second, one can simulate replicate time series to account for the inherent stochasticity of disease transmission, so that conclusions are not based on one-off events that might result in confirmation bias [24]. Third, we can specifically simulate re-emergence and elimination events to separate the stress testing of EWS from the research necessary to operationalize EWS. In short, a simulation approach to stress testing provides the flexibility of a theoretical model, but remains tethered to reality because the model parameters are fitted to real data. We believe this is the first study to take this step towards operationalizing EWS as part of infectious disease early warning systems.

Here, we report on a study using simulations from fitted models of measles transmission in an outbreak-prone population to test whether CSD anticipates critical transitions in realist situations. We focus on two scenarios: the re-emergence of measles following a large outbreak, a situation typical of measles dynamics in the Sahel [25], and the elimination of measles by gradually increasing routine vaccination. We seek to answer two related questions. First, can CSD distinguish between time series of disease incidence when the underlying dynamics are far from versus near to a critical transition? Second, can CSD anticipate disease re-emergence and elimination?

To answer these questions, we fit mechanistic models to time series of measles incidence in four cities in Niger [25,26]. We then use the fitted models to perform simulation experiments designed to test the performance of several EWS that are characteristic of CSD with respect to anticipating re-emergence and elimination. Our results confirm the theory concerning several EWS. In particular, we show that slowing down before a critical transition is detectable by several EWS in realistic scenarios. However, our study also highlights the limitations of EWS in situations where disease re-emergence and elimination occur rapidly. Finally, we also find that EWS perform better at anticipating re-emergence than elimination.

## 2. Material and methods

### 2.1. Data

**Figure 1.**
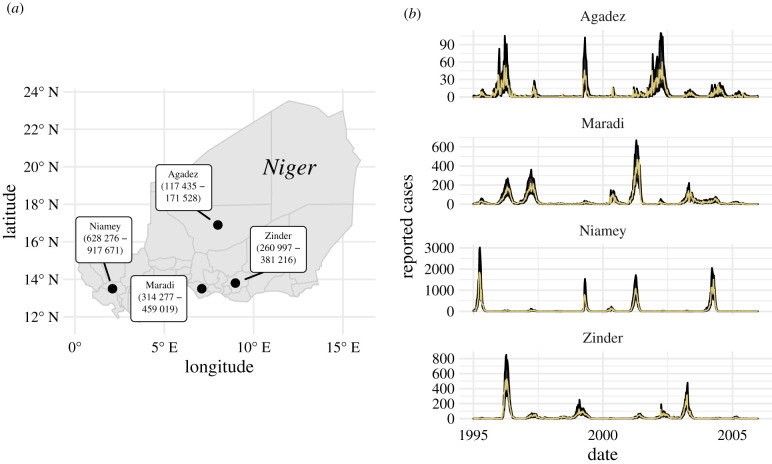
Locations of data sources and observed and predicted measles dynamics. (*a*) Locations and 1995–2005 population-size ranges (in parentheses) of our four focal cities in Niger. (*b*) Time series of weekly reported cases (incidence data; yellow solid lines) and the 68% prediction intervals (black ribbons) for one-week-ahead predictions from our fitted susceptible-exposed-infected-recovered (SEIR) models for each city.

We used weekly measles case report data (incidence) from four Nigerien cities: Agadez, Maradi, Niamey and Zinder (figure 1*a*). The data were collected over an 11-year period from 1995 to 2005 (figure 1*b*). These data are ideal for testing the theory of CSD in disease dynamics because each city has a different population size (with means ranging from about 150 000 to 750 000 during this time period), different dynamics in terms of outbreak sizes (maximum weekly incidence ranging from 60 to 1845 cases) and length of inter-epidemic periods (2–5 years), and has different amounts of demographic stochasticity due to differences in population size. Such differences provide an interesting test case for EWS because different levels of demographic stochasticity due to population sizes and environmental stochasticity due to potential differences in transmission dynamics can influence CSD [27–30]. We also used data on district population sizes and national-level birth rates (electronic supplementary material, figure S1). Measles incidence data and district-level population data were obtained from the Niger Ministry of Health [31]. National-level birth rate data were downloaded from the FRED database (https://fred.stlouisfed.org/series/SPDYNCBRTINNER).

### 2.2. Stochastic susceptible-exposed-infected-recovered model

The model is a discrete-time approximation to the continuous-time susceptible-exposed-infected-recovered (SEIR) model with demography, specified as a set of difference equations,

2.1 $$S_{t+\Delta t}-S_{t}=n_{0S,t}-n_{SE,t}-n_{S0,t,}$$

2.2 $$E_{t+\Delta t}-E_{t}=n_{SE,t}-n_{EI,t}-n_{E0,t,}$$

2.3 $$I_{t+\Delta t}-I_{t}=n_{EI,t}+n_{0I,t}-n_{IR,t}-n_{I0,t}$$

2.4 $$\;\mathrm{and}\;R_{t+\Delta t}-R_{t}=n_{0R,t}+n_{IR,t}-n_{R0,t},$$

where **n**_*t*_ are random variables representing the number of individuals transitioning into or out of each class at each update *t* → *t* + Δ*t*. Transition definitions are in table 1.

**Table 1.** Transitions in the simulating model.

random variable	transition	(Δ*S*, Δ*E*, Δ*I*, Δ*R*)
*n*_0*S*_	births into the *S* compartment, not vaccinated	(1, 0, 0, 0)
*n*_*SE*_	number of people transitioning from *S* to *E*	(−1, 1, 0, 0)
*n*_*S*0_	number of deaths leaving *S*	(−1, 0, 0, 0)
*n*_*EI*_	number of people transitioning from *E* to *I*	(0, −1, 1, 0)
*n*_*E*0_	number of deaths leaving *E*	(0, −1, 0, 0)
*n*_0*I*_	number of imported infections	(0, 0, 1, 0)
*n*_*IR*_	number of people transitioning from *I* to *R*	(0, 0, −1, 1)
*n*_*I*0_	number of deaths leaving *I*	(0, 0, −1, 0)
*n*_0*R*_	births into the *R* compartment, vaccinated	(0, 0, 0, 1)
*n*_*R*0_	number of deaths leaving *R*	(0, 0, 0, −1)

The stochastic random variables are specified as follows:

2.5 $$n_{0S,t}∼\text{Poisson}\;((1-p_{t})\mu N\times \Delta t),$$

2.6 $$(n_{SE,t},n_{S0,t})∼\text{EulerMultinomial}\;(S_{t},(\beta_{t}I_{t}/N,\nu ),\Delta t),$$

2.7 $$(n_{EI,t},n_{E0,t})∼\text{EulerMultinomial}\;(E_{t},(\eta ,\nu ),\Delta t),$$

2.8 $$(n_{IR,t},n_{I0,t})∼\text{EulerMultinomial}\;(I_{t},(\gamma ,\nu ),\Delta t),$$

2.9 $$n_{I0,t}∼\text{Poisson}\;(\psi \times \Delta t),$$

2.10 $$n_{0R,t}∼\text{Poisson}\;(p_{t}\mu N\times \Delta t)$$

2.11 $$\;\mathrm{and}\;n_{R0,t}∼\text{EulerMultinomial}\;(R_{t},(\nu ),\Delta t),$$

where *p*_*t*_ is the vaccination probability, *μ* is the *per capita* birth rate at time *t*, *N* is population size at time *t*, ∼EulerMultinomial (*T*, *r*_*i*_, Δ*t*) specifies that the variables on the left of ∼ follow a Euler multinomial distribution for *T* individuals with hazard rates *r*_*i*_ and step size Δ*t*, *β*_*t*_ is a time-varying rate of transmission, *η* is a time-invariant rate of transfer from the exposed class to the infectious class, *γ* is a time-invariant recovery rate, *ν* is the *per capita* death rate, and *ψ* is the rate of imported infections (estimated by the model). The Euler multinomial distribution corresponds to a multinomial distribution where the event probabilities *P*_*i*_ are determined by the Euler time step Δ*t* and the hazard rates *r*_*i*_, according to,

2.12 $$P_{0}=\exp\left(-\sum_{i}r_{i}\Delta t\right)$$

and

2.13 $$P_{i}=\frac{(1-P_{0})r_{i}}{\left(\sum_{i}r_{i}\right)}\;\text{for}\;i>0.$$

Event zero is the event that an individual stays in its initial compartment and does not contribute to the vector **n**_*t*_. To provide a specific example, equation (2.7) corresponds to,

2.14 $$\begin{array}{rl} & \left(E_{t}-n_{EI,t}-n_{E0,t},n_{EI,t},n_{E0,t}\right)\\ & \;∼\text{Multinomial}(E_{t};P_{0},\frac{(1-P_{0})\eta }{\left(\eta +\nu \right)},\frac{(1-P_{0})\nu }{\left(\eta +\nu \right)}), \end{array}$$

where *P*_0_ = exp (− (*η* + *ν*)Δ*t*). We used a daily time step of Δ*t* = year/365.

We modelled the rate of transmission as

2.15 $$\beta_{t}=\beta \left(1+\exp\left(\sum_{i=1}^{6}q_{i}\xi_{i_{t}}\right)\right)\Gamma_{t}.$$

where *β* is the minimum transmission rate over the season and the term $\sum_{i=1}^{6}q_{i}\xi_{i_{t}}$ is a B-spline to model seasonality in transmission. The B-spline bases ($\xi_{i_{t}}$) are periodic with a 1-year period. The transmission rate (*β*_*t*_) is also subject to stochastic process noise at each time step, $\Gamma_{t}$, which we model as gamma-distributed white (temporally uncorrelated) noise with mean 1 and intensity *σ*^2^ [32]. In this model, the effective reproduction number at time *t* may be approximated as: $R_{e}(t)\approx (\beta_{t}/\gamma )(S_{t}/N_{t})$. The effect of vaccination on $R_{e}(t)$ is implicitly included through the size of the *S*_*t*_ compartment.

Observed case reports (*y*_*t*_) were drawn from a negative binomial distribution subject to a constant reporting fraction (*ρ*) and dispersion parameter *τ*,

2.16 $$y_{t}∼\text{Negative Binomial}\;(\rho x_{t},\tau ),$$

where *x*_*t*_ are the accumulated cases that transition from the infected class to the recovered class in a one-week period. In this parametrization of the negative binomial, the mean is equal to *ρx*_*t*_ and the variance is equal to *ρx*_*t*_ + (*ρx*_*t*_)^2^*τ*.

### 2.3. Model fitting and inference

The model described above was fit to the time series of case reports (incidence data) from each city using maximization by iterated particle filtering (MIF) [33]. We estimated 14 parameters (table 2). To improve parameter identifiability, mean incubation period was fixed to 1/η=8 days and the mean infectious period was set to 1/γ=5 days [34]. The vaccination probability (*p*_*t*_) was set to 70% for all times *t*, consistent with reported vaccination coverage [25].

**Table 2.** Model parameters, definitions and indicator as to whether they were fitted or fixed. Sources for fixed values are cited in the main text.

parameter symbol	definition	fitted or fixed
*β*	minimum transmission rate within the season	fitted
*q*_*i*_	seasonal transmission spline parameters (i∈1,2,3,…, 6)	fitted
*S*_(*t*=0)_/*N*_*t*=0_	initial susceptible fraction	fitted
*E*_(*t*=0)_/*N*_*t*=0_	initial exposed fraction	fitted
*I*_(*t*=0)_/*N*_*t*=0_	initial infected fraction	fitted
*ψ*	importation rate	fitted
*ρ*	reporting fraction	fitted
*σ*	gamma white-noise intensity	fitted
*τ*	negative binomial dispersion	fitted
1/*η*	incubation period	fixed (8 days)
1/*γ*	infectious period	fixed (5 days)
*p*_*t*_	vaccination probability	fixed (0.7)
*mu*_*t*_	*per capita* birth rate	fixed
*N*_*t*_	population size	fixed

For model fitting, we made simplifying assumptions to make the estimation procudure more tractable. First, we used known population size (*N*_*t*_) for each city in each year. Second, death (*ν*) was not included in the model when fitting because the rate of infection is much faster than the rate of death and because we used known population size at each time step. Excluding deaths means we can avoid making further assumptions about demography. This means we ignored the *R* compartment entirely for model fitting.

MIF relies on particle filtering, which estimates the likelihood of fixed parameters by integrating state variables of a stochastic system. To identify the maximum-likelihood estimates (MLEs), MIF lets parameters take a random walk during the filtering process and selectively propagates forward parameter sets (i.e. particles) with the highest likelihood. The variance of the random walk decreases at each iteration of MIF, where a MIF iteration means one filtering pass through the time series. This procedure converges toward the MLEs.

We used the iterated filtering 2 (IF2) algorithm [33] implemented in the R [35] package pomp v. 1.18 [36,37] to conduct MIF. To initialize MIF, we generated 5000 parameter sets using Latin hypercube sampling over large ranges of the parameters. We then performed two rounds of MIF, each for 100 iterations, with 10 000 particles, using geometric cooling. For the first round of MIF, we set cooling.factor=1. For the second round, which was initialized using the collection of parameter sets from the end of the first round, we set cooling.factor=0.9. We computed the log-likelihood of 5000 final MIF parameter sets (i.e. parameter sets collected after 200 MIF iterations) as the log of the mean likelihoods of 50 replicate particle filters with 10 000 particles each. At this stage, we took the parameter set with highest log-likelihood to be the MLE.

We used the parametric bootstrap to estimate approximate 95% confidence intervals for all parameters, conducted for each city independently, as follows. First, we simulated 100 realizations from the fitted model using the MLE parameters. Second, we fitted the SEIR model to each of the 100 bootstrap simulations using the same MIF procedure described above, except we initiated the parameter search from 50 parameter sets rather then 5000. We reduced the number of parameter sets due to the excessive computing time required to fit 100 simulated datasets for each of the four cities. Third, we identified the MLE parameter set for each of the 100 bootstrap simulations from among the 50 MIF parameter sets. Last, we calculated summary statistics (mean, median, quantiles) from the distribution of 100 MLE parameters.

### 2.4. Model assessment

We used the MLE parameter sets to make one-week-ahead predictions and compared observed and expected case counts. To make one-week-ahead predictions, we used particle filtering with 50 000 particles and retained the mean and standard deviation of all latent states across all particles before they were filtered at each time step. We used the mean predictions ($E({\text{cases}}_{t})$) to assess model fit using a generalized coefficient of determination, calculated as: $R^{2}=1-(\sum_{t}{\left[E({\text{cases}}_{t})-{\text{cases}}_{t}\right]}^{2}/\sum_{t}{\left[\text{mean( cases) }-{\text{cases}}_{t}\right]}^{2})$ [38].

In addition to comparing model expectations with in-sample observed data, we also compared our fitted SEIR models with two benchmarks: a negative binomial sampling model that assumes independent and identically distributed observations and a seasonal autoregressive moving average (SARIMA) model. Doing so allows us to gain some intuition as to whether accounting for mechanism (i.e. transmission dynamics) improves model fit and inference. The SARIMA is a seasonal autoregressive moving average model (an $\mathrm{ARIMA}\;(2,0,2){\left(1,0,1\right)}_{52}\;\mathrm{model}$) that can account for data dependencies and annual periodicity. Outperforming the SARIMA is a harder test than outperforming the negative binomial sampling model. We fitted both models to the weekly case count observations for each city using maximum likelihood. After fitting, we calculated Akaike’s information criterion for each model as: AIC = 2*k* − 2ln(*L*), where *k* is the number of estimated parameters in the model and *L* is the model’s likelihood [39]. The negative binomial sampling model has two parameters, the SARIMA model has eight parameters, and the SEIR model has 14 parameters.

### 2.5. Model simulations

To fit the SEIR model, we used known population size interpolated between years. This meant we were able to ignore certain demographic processes. For example, deaths from the susceptible pool were ignored under the assumption that the infection rate was much faster than the death rate. We also ignored the recovered class because their dynamics, outside of the contribution to population size (which was assumed known), do not impact the *S*, *E* or *I* compartments. However, births and deaths from all compartments, including *R*, were needed when simulating the model over arbitrarily long time periods that do not necessarily represent real times for which we would have information on population size. For long-run simulations, we set *μ* = *ν* = 0.05, which is the *per capita* rate reported for 2005, rounded to the nearest 0.01. Setting birth rate equal to death rate achieved an equilibrium population size for long-run simulations.

#### 2.5.1. Simulating re-emergence

To simulate re-emergence of measles, we manipulated the initial size of the susceptible pool to represent an increase from low $R_{e}(t)$ to high $R_{e}(t)$. Doing so allowed us to test whether EWS can distinguish between windows of time when $R_{e}(t)$ is far from a critical transition and when $R_{e}(t)$ is near a critical transition. We reduced the initial fraction of susceptible individuals by multiplying the MLE for *S*_(*t*=0)_ by six depletion factors: 1 × 10^−4^, 0.1, 0.2, 0.3, 0.4 and 0.5. These depletion factors represent situations of susceptible depletion after outs of various size. After defining *S*_(*t*=0)_ based on the depletion factor, we then set the initial number of recovered individuals to *R*_(*t*=0)_ = *N*_(*t*=0)_ − *S*_(*t*=0)_ and set the initial number of exposed and infected individuals to zero. Initial population size for simulation scenarios, *N*_(*t*=0)_, was set to the mean population size for each city over the 1995–2005 time period. We then simulated the model forward for 40 years using the mean birth rate for the entire country (*μ* = 0.05) and setting the death rate equal to the birth rate (*μ* = *ν* = 0.05) to achieve a constant average total population size over the course of the simulation (total population size does vary, though, because of stochasticity in the model). Forty years was long enough for $R_{e}(t)$ to reach or exceed 1 for each simulation replicate. Several outs are seen within 11 years in the data (figure 1*b*), though time-to-outbreak was larger in scenarios where the susceptible population was initialized to be much smaller than ever observed in reality. Because the model is stochastic, we repeated these simulations 500 times for each city–susceptible-depletion combination.

Next, we split each simulated time series into null and test intervals. Fixed-size windows before (null) and after (test) the known critical transition were used because our focus was on testing EWS using empirically based models, rather than attempting to identify optimal methods for operationalizing EWS. To define the null and test intervals for our simulations of re-emergence and elimination, we need to know when the critical transition between alternative modes of fluctuation occurs. For re-emergence, we defined the critical year as the year in which the effective reproduction number ($R_{e}$) reaches or exceeds the critical value of 1. When determining the critical year, we eliminated the variation in $R_{e}$ that arises from the gamma white noise factor and thus calculated the expected value of $R_{e}(t)$ over the set of all simulation replicates at time *t*. From a window ranging from the beginning of the simulation to the end of the critical year, we defined the null interval as the first half of the window (far from $R_{e}=1$) and the test interval as the second half of the window (near $R_{e}=1$). We did this for each city and for each level of susceptible depletion, and calculated EWS over all null and test intervals separately.

#### 2.5.2. Simulating elimination

To simulate elimination, we simulated an improvement in routine vaccination in which the vaccination probability increased linearly with respect to time to eventually reach 100%, i.e. eradication (electronic supplementary material, figure S3). Vaccination probablity started at baseline vaccine coverage reported for Niger of 70%, *p*_*t*_ = 0.7 [25] and simulations were initialized at the MLE for *S*_(*t*=0)_.

We defined the critical time as the year in which the vaccination probability reaches the threshold needed for herd immunity. This vaccination threshold is defined as $p^{*}=1-1/R_{0}$. Because our transmission function is seasonal, we first approximated time-specific $R_{0}$ as: $R_{0}(t)=\frac{\eta \beta_{t}}{\left(\eta +\nu )(\gamma +\nu \right)}$, where 1/*η* is the incubation period, 1/*γ* is the infectious period, *β*_*t*_ is the time-specific rate of transmission, and *ν* is the death rate. We took a conservative approach for calculating the vaccination threshold by using the maximum value of $R_{0}(t)$ out of the range of values in its seasonal variation. That is, we used $p^{*}=1-1/\text{max}(R_{0}(t))$ as the threshold value. We set the time at which the vaccination probability is equal to this threshold value as the endpoint for the EWS analysis. All elimination simulations had vaccination improvements that started at year 50. Therefore, we defined the test interval as the times between the beginning of year 50 and the time at which the vaccination probability is equal to p*. We then defined the null interval as a window with length equal to the test interval and ending at the end of year 49. EWS were then calculated for each interval.

### 2.6. Calculating early warning signals

We considered eight candidate EWS based on previous work [4,13–15,18] (table 3). We used the spaero::get_stats() function [40] in R [35] to calculate EWS according to the formulae in table 3. All EWS except the coefficient of variation are expected to increase as $R_{e}(t)$ approaches 1 from below [4,13,14]. We are not aware of theoretical results for the behaviour of these EWS as $R_{e}(t)$ approaches 1 from above that are applicable to our fitted model’s dynamics, which are highly nonlinear (electronic supplementary material, figure S3). But a natural expectation is that the mean should decrease as the endemic equilibrium of our model’s deterministic skeleton moves towards zero.

**Table 3.** List of candidate early warning signals and their estimating equations. See [15] for details.

EWS	estimator	theoretical correlation with $R_{e}(t)$ in an emergence scenario
mean	$\bar{y}=\sum_{i=1}^{n}\frac{y_{i}}{n}$	positive
variance	$s^{2}=\sum_{i=1}^{n}\frac{{\left(y_{i}-\bar{y}\right)}^{2}}{n}$	positive
coefficient of variation	$\frac{s}{\bar{y}}$	null
index of dispersion	$\frac{s^{2}}{\bar{y}}$	positive
skewness	$\frac{1}{s^{3}}\sum_{i=1}^{n}\frac{{\left(y_{i}-\bar{y}\right)}^{3}}{n}$	positive
kurtosis	$\frac{1}{s^{4}}\sum_{i=1}^{n}\frac{{\left(y_{i}-\bar{y}\right)}^{4}}{n}$	positive
autocovariance	$s_{1}^{2}=\sum_{i=2}^{n}\frac{\left(y_{i}-\bar{y})(y_{i-1}-\bar{y}\right)}{n}$	positive
autocorrelation	$r_{1}=\frac{s_{1}^{2}}{s^{2}}$	positive

For each simulation of re-emergence and elimination, we calculated EWS for the time series of expected cases in the null and test intervals. This yielded a distribution of EWS over the 500 null and test intervals. We assessed the performance of each EWS using the area under the curve (AUC) statistic. Specifically, we used AUC to calculate the amount of overlap between the distributions of each EWS from the null and test intervals [15]. Values of AUC far from 0.5 (i.e. close to 0 or 1) indicate a greater degree of separation and thus better performance of a particular EWS in terms of classifying whether $R_{e}(t)$ is close to a critical transition. We calculated AUC as: AUC = [*r*_test_ − *n*_test_ (*n*_test_ + 1)/2]/(*n*_test_*n*_null_) where *r*_test_ is the sum of the ranks of test set EWS statistics in a combined set of null and test statistics (lower numbers have lower ranks), *n*_test_ is the number of test of statistics and *n*_null_ is the number of null statistics [15,41]. The AUC of an EWS is the probability that a randomly chosen EWS value from the test set is higher than an EWS value randomly chosen from the null set [42]. Therefore, AUC should be high (closer to 1) when an EWS is expected to increase as a critical transition is approached, whereas AUC should be low (closer to 0) when an EWS is expected to decrease.

## 3. Results

**Figure 2.**
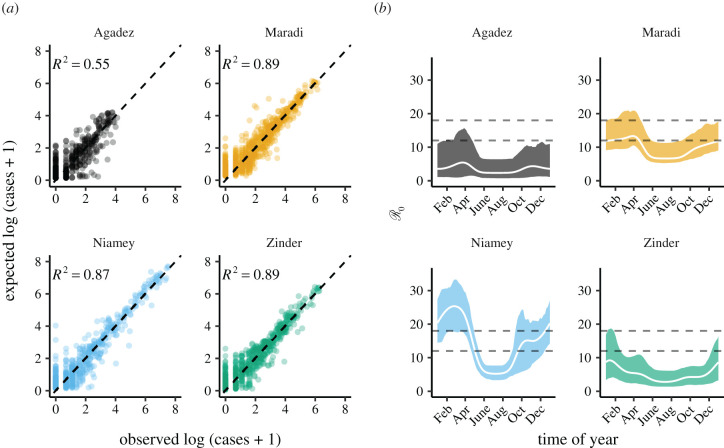
Accuracy of the fitted SEIR models and estimated seasonality. (*a*) Comparison of in-sample model predictions and observations for each city. Expected cases are one-week-ahead predictions from the fitted models. The dashed line shows 1 : 1. Coefficients of determination (*R*^2^) were calculated as the reduction in the sum-of-squared errors from model predictions relative to a null model of the mean number of cases (Material and methods). (*b*) The estimated seasonality of the basic reproductive ratio ($R_{0}$) for each city. $R_{0}$ was approximated as: *ηβ*_*t*_/((*η* + *ν*)(*γ* + *ν*)), where 1/*η* is the incubation period, 1/*γ* is the infectious period, *β*_*t*_ is the time-specific rate of transmission, and *ν* is the death rate. Only *β*_*t*_ is estimated by our model. We set 1/η=8 days, 1/γ=5 days, and *ν* = 0.05 for calculating $R_{0}$ as shown in this figure. The white line is $R_{0}$ calculated using the MLE parameters; shaded regions are the bootstrapped 95% confidence intervals. The dashed horizontal lines show the common range of measles $R_{0}\;:\;12\;\mathrm{to}\;18$.

The fitted models adequately reproduce observed dynamics (figure 1*b*), with in-sample *R*^2^s from one-week-ahead predictions ranging from 0.55 for Agadez to 0.89 for Maradi (figure 2*a*). The fitted models also had lower AIC values than two benchmarking models (table 4). The estimated seasonality is consistent with previously estimated patterns, including the decline in seasonality amplitude as population size decreases (figure 2*b*) [25]. Our estimates of $R_{0}$ do not all perfectly overlap with the often-cited range of 12–18 (figure 2*b*) [43], but more recent reviews suggest that measles’ $R_{0}$ is much more variable and is context-specific [44].

**Table 4.** AIC values for the benchmarking and SEIR models.

city	neg. binomial	SARIMA	SEIR
Agadez	2463	2010	1949
Maradi	4618	3547	3521
Niamey	4112	3185	2937
Zinder	3958	2902	2859

Our model for Agadez performed poorly relative to the other cities, but still did better than non-mechanistic models (table 4). MLE and bootstrapped 95% confidence intervals for all parameters are in the electronic supplementary material, tables S1–S4. Parameter correlations were generally weak (electronic supplementary material, S3), but the correlation between initial susceptible population size (*S*_*t*=0_/*N*_*t*=0_) and transmission rate (*β*) was negative and larger than −0.5 for all cities. Overall, weak parameter correlations indicated high parameter identifiability in the model.

**Figure 3.**
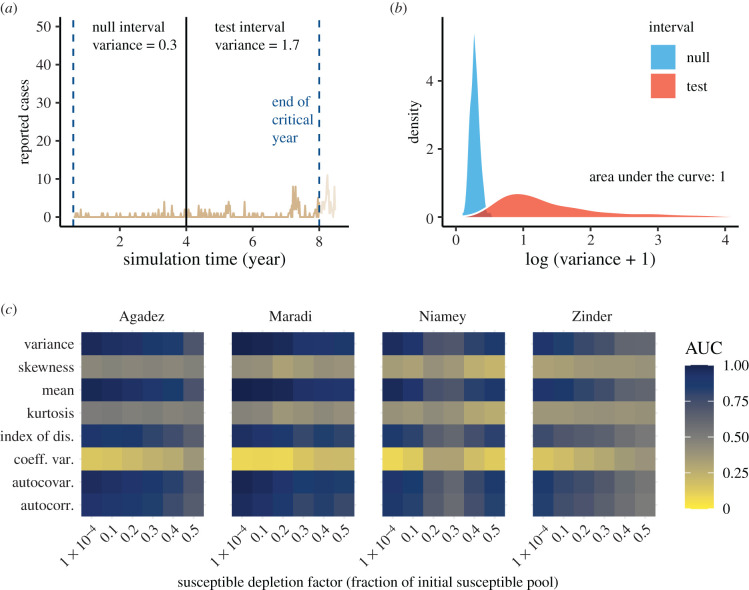
Performance of early warning signals (EWS) over fixed windows on the approach to emergence. (*a*) A typical example of an emergence simulation for Maradi. The two vertical blue lines indicate the start (left-most line) and end (line for critical year) of the full window. The black line demarcates the division between the equal-length null and test intervals, in which we show the calculated variance. (*b*) Empirical densities of variance in the null and test intervals across 500 simulations and the associated area under the curve (AUC) statistic. (*c*) Heatmap of AUC statistics for each EWS at each level of susceptible depletion factor. AUC values closer to 0 or 1 indicate higher ability to distinguish among time series near and far from a critical transition. See electronic supplementary material, figure S8 for a visualization of how susceptible depletion factor maps to number of weeks in the null and test intervals.

The EWS generally performed as predicted by theory with respect to the approach to re-emergence. Most EWS increased as the critical transition was approached, resulting in AUC values above 0.5 and often near 1 (figure 3). Skewness, kurtosis and coefficient of variation performed poorly across all levels of susceptible depletion in all cities.

Variance, mean, index of dispersion, autocovariance and autocorrelation all performed equally well at predicting re-emergence (figure 3*c*). Their performance declined as the size of the susceptible pool increased (i.e. a larger susceptible depletion factor). This is expected because a larger susceptible pool results in more rapid returns to $R_{e}(t)=1$, which, in turn, results in shorter null and test intervals, making estimates of EWS less precise [14]. Thus, re-emergence may prove difficult to anticipate in ‘fast’ transmission systems, as observed in simulations in [14] and seen here when susceptible depletion was relatively small (figure 3*c*).

**Figure 4.**
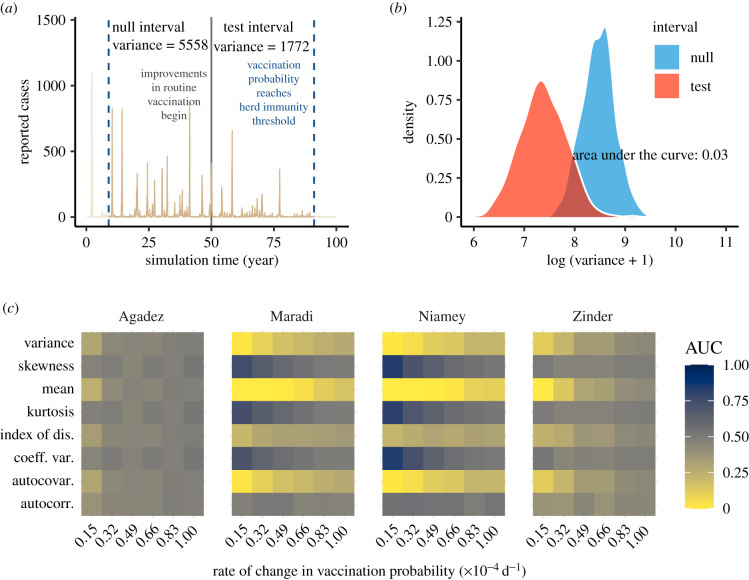
Performance of early warning signals (EWS) over fixed windows on the approach to elimination. (*a*) A typical example of an elimination simulation for Maradi. The two vertical blue lines indicate the start (left-most line) and end (line for critical year) of the full window. The black line demarcates the division between the equal-length null and test intervals, in which we show the calculated variance. (*b*) Empirical densities of variance in the null and test intervals across 500 simulations and the associated area under the curve (AUC) statistic. (*c*) Heatmap of AUC statistics for each EWS at each speed of approach to herd immunity. AUC values closer to 0 or 1 indicate higher ability to distinguish among time series near and far from a critical transition. See electronic supplementary material, figure S8 for a visualization of how vaccination speed maps to number of weeks in the null and test intervals.

The EWS performed less well when anticipating elimination, relative to emergence (figure 4). Only three metrics were reliable: mean, autocovariance and variance. All three metrics decreased as $R_{e}(t)$ approached the critical transition (electronic supplementary material, figure S6). As in the case of anticipating elimination, AUC values moved closer to 0.5 as the rate of vaccination increased (figure 4*c*).

## 4. Discussion

The ability to detect early warnings of outbreaks of potentially fatal diseases such as measles during non-epidemic periods of unpredictable duration may facilitate planning such as enhanced surveillance to expedite outbreak detection, implementation of serological surveys to identify immunity gaps, and initiation of targeted supplemental vaccination [45,46]. Further, it has been argued that measles eradication requires consideration of local demographic factors [47] and that regional outbreak response vaccination can be effective when initiated early, even if coverage is suboptimal [45]. Consequently indicators that a location or a region is on a path to outbreak or elimination can help to prioritize the timing and distribution of limited resources. Using empirically based transmission models, we found that generic indicators of CSD were informative regarding simulated re-emergence and elimination of measles. Our conclusions are, of course, dependent on the modelling choices we made, but using empirically based models represents a step in the progression from theory to simulations to applied science. This work fills the important gap between data-free simulations and application of EWS as part of a decision-support toolkit.

Overall, generic indicators performed better in scenarios of re-emergence compared with elimination. Moreover, indicators behaved as expected based on simple one-dimensional models of fluctuations around an equilibrium with gradually declining stability—the prototypical model of CSD. This is because the autocorrelation and variance increased on the approach to $R_{e}=1$ (figure 3*c*). If, alternatively, the variance had increased but the autocorrelation had not, a better-supported model would be an equilibrium with fixed stability but subject to increasing intensity of perturbations. From a model selection point of view, our simulations of re-emergence support the prototypical model of CSD. Since our simulations were fit to measles data and had relatively high one-step-ahead predictive accuracy on average, it suggests that the prototypical model of CSD may be supported by datasets similar to the ones we fit and can provide predictive value for them. The definitive test of this approach, or course, would be to record forecasts of re-emergence based on indicators of CSD and evaluate their accuracy as data are accumulated. A recent study using a different method for detecting critical transitions found that elimination was easier to detect than re-emergence, contrary to our findings [48]. This suggests different approaches could be considered for different applications.

Kurtosis and skewness did not perform well (AUC far from 0 and 1). This may be because higher moment-based indicators may need longer time series and higher amounts of stochasticity to detect critical transitions. Thus, while kurtosis and skewness were not strong indicators of the transition to re-emergence in our system, they may still prove useful in other systems.

We found lag-1 autocorrelation not to be a strong indicator of the transition to elimination (figure 4*c*). Thus, the prototypical model of CSD appears less useful in this scenario. Other EWS (variance, autocovariance and mean) did show altered behaviour as the transition to elimination was approached, but these EWS were less sensitive under elimination scenarios compared with re-emergence scenarios. These results suggest that although distributional changes in indicators occur prior to disease elimination, interpreting these changes as the loss of stability of an endemic equilibrium, or declining $R_{e}$, requires a model which accounts for complicating system features such as seasonality, nonlinearity, damped oscillations and local extinction. We also found that EWS in smaller cities performed worse relative to larger cities at predicting elimination (figure 4*c*). Our hypothesis is that stochastic effects are stronger in smaller populations, which introduces more variation into our EWS and makes trends more difficult to identify. Spatial replication across small populations, which should reduce stochasticity, could prove important for developing practical EWS.

A potential limitation of our findings is that the susceptible depletion factors in our simulation study (figure 3*c*) might be smaller than the factors that occur in reality. A small depletion factor corresponds to a large level of susceptible depletion. To check the relevance of this concern, we calculated the level of susceptible depletion after outbreaks (defined as years where the total number of cases reached 80% of the maximum observed) across 100 replicate simulations (electronic supplementary material). We found that susceptible depletion was less than 0.5, the smallest susceptible depletion level we tested, for 0.9% of outbreaks in Agadez, 21% of outbreaks in Maradi, 100% of outbreaks in Niamey and 26% of outbreaks in Zinder. These statistics do not detract from our main finding of CSD in measles dynamics, but do suggest that EWS might be less useful in some cases than in others. For example, AUC values for emergence at the 0.5 level of susceptible depletion were already low for most cities (figure 3*c*). Thus, our methods may not be practical for cities that rarely experience levels of susceptible depletion below 0.5 (e.g. Agadez).

Another potential limitation is that the population of Niger has increased substantially since the end of the time series we analysed in 2005. This presents a limitation to our work because we cannot definitively say whether our results are robust to such large changes in population size. However, our analysis across cities with different population sizes suggests that EWS would remain a viable tool. Elimination may be easier to detect, as demonstrated by our result that EWS performed better in larger cities.

We focused on fixed windows for calculating EWS based on the timing of the critical transition. Thus, our work also does not represent how EWS would be operationalized in online mode. The fixed windows were specifically designed to test whether EWS could detect a known critical transition and limit the additional complexity associated with choosing a moving window size. Determining the optimal moving window width is non-trivial [18] and is beyond the scope of this article. Nonetheless, our analyses show that several EWS can detect critical transitions in representatively noisy SEIR dynamics. Providing this link from theory to application through empirically based models represents an important step towards operationalizing EWS. Further applied work on optimal moving window widths and the lead-time of different EWS appears to be worth the effort given our encouraging results at the theoretical–applied divide. This is especially true because fitting even relatively simple models of disease transmission for a well-known disease such as measles is difficult. The ability to rely on model-free indicators of critical transitions therefore remains an important endeavour, especially for emerging pathogens.

Unpredictable, recurring outbreaks with seasonality in transmission such as those observed for measles in Niger during 1995–2005 are challenging settings for the application of EWS in part because most theory regarding the behaviour of EWS [14,30] is based on models that exhibit simpler dynamics. Consequently, the development of robust, model-independent early warning systems for infectious diseases [1] probably will benefit from further study of the behaviour of EWS in complex models. Also, although we have shown that CSD precedes tipping points in stochastic models that were fit to data with state-of-the-art methods, how to operationalize the phenomenon of CSD remains an open research area [49]. Emerging technologies like artificial intelligence might offer new ways to find optimal detection thresholds for EWS [18]. Therefore, EWS, which provide timely insight and are now accompanied with additional support, could become a key part of a decision-support toolkit.

## Data Availability

Codes and data needed to reproduce all analyses in this manuscript have been archived on Zenodo: https://doi.org/10.5281/zenodo.6678151 [50]. The data are provided in the electronic supplementary material [51].
